# Bulk and single-cell RNA-sequencing analyses along with abundant machine learning methods identify a novel monocyte signature in SKCM

**DOI:** 10.3389/fimmu.2023.1094042

**Published:** 2023-05-25

**Authors:** Yuyao Liu, Haoxue Zhang, Yan Mao, Yangyang Shi, Xu Wang, Shaomin Shi, Delin Hu, Shengxiu Liu

**Affiliations:** ^1^ Department of Burns, The First Affiliated Hospital of Anhui Medical University, Hefei, Anhui, China; ^2^ Department of Dermatovenerology, The First Affiliated Hospital of Anhui Medical University, Hefei, Anhui, China; ^3^ Key Laboratory of Dermatology, Ministry of Education, Hefei, Anhui, China; ^4^ Inflammation and Immune Mediated Diseases Laboratory of Anhui Province, Anhui Medical University, Hefei, Anhui, China; ^5^ Department of Dermatology, The Third Hospital of Hebei Medical University, Shijiazhuang, Hebei, China; ^6^ Department of Emergency Surgery, The First Affiliated Hospital of Anhui Medical University, Hefei, Anhui, China; ^7^ Department of General Surgery, The First Affiliated Hospital of Anhui Medical University, Hefei, Anhui, China

**Keywords:** skin cutaneous melanoma, single-cell, tumor immune microenvironment, monocyte, machine learning, IFITM3

## Abstract

**Background:**

Global patterns of immune cell communications in the immune microenvironment of skin cutaneous melanoma (SKCM) haven’t been well understood. Here we recognized signaling roles of immune cell populations and main contributive signals. We explored how multiple immune cells and signal paths coordinate with each other and established a prognosis signature based on the key specific biomarkers with cellular communication.

**Methods:**

The single-cell RNA sequencing (scRNA-seq) dataset was downloaded from the Gene Expression Omnibus (GEO) database, in which various immune cells were extracted and re-annotated according to cell markers defined in the original study to identify their specific signs. We computed immune-cell communication networks by calculating the linking number or summarizing the communication probability to visualize the cross-talk tendency in different immune cells. Combining abundant analyses of communication networks and identifications of communication modes, all networks were quantitatively characterized and compared. Based on the bulk RNA sequencing data, we trained specific markers of hub communication cells through integration programs of machine learning to develop new immune-related prognostic combinations.

**Results:**

An eight-gene monocyte-related signature (MRS) has been built, confirmed as an independent risk factor for disease-specific survival (DSS). MRS has great predictive values in progression free survival (PFS) and possesses better accuracy than traditional clinical variables and molecular features. The low-risk group has better immune functions, infiltrated with more lymphocytes and M1 macrophages, with higher expressions of HLA, immune checkpoints, chemokines and costimulatory molecules. The pathway analysis based on seven databases confirms the biological uniqueness of the two risk groups. Additionally, the regulon activity profiles of 18 transcription factors highlight possible differential regulatory patterns between the two risk groups, suggesting epigenetic event-driven transcriptional networks may be an important distinction. MRS has been identified as a powerful tool to benefit SKCM patients. Moreover, the IFITM3 gene has been identified as the key gene, validated to express highly at the protein level via the immunohistochemical assay in SKCM.

**Conclusion:**

MRS is accurate and specific in evaluating SKCM patients’ clinical outcomes. IFITM3 is a potential biomarker. Moreover, they are promising to improve the prognosis of SKCM patients.

## Introduction

Skin cutaneous melanoma (SKCM) is the most aggressive type of all skin malignancies. Worldwide, an increase in the morbidity of SKCM has particularly raised alarm (1). For localized lesions, surgery is the most recommended treatment modality, perfectly able to assure wound healing, and is warranted in all stages of the disease (2, 3). Once the aggressive dissemination happens, other forms of therapies (chemotherapy, immunotherapy, targeted therapy and radiotherapy, or integrated combinations of them) must be provided simultaneously (4, 5). However, the overall prognosis of SKCM patients still stays poor (6) due to drug resistance, distant metastasis and high recurrence rate, etc. Therefore, more specific molecular biomarkers with prognostic and therapeutic significance are required.

In solid cancer, tumor microenvironment (TME) has been reckoned as an important structure. TME encompasses multiple cell types (stromal cells, fibroblasts, endothelial cells, innate and adaptive immune cells, etc.) and extracellular components (growth factors, cytokines, extracellular matrix, hormones, etc.) that surround cancerous cells (7). TME co-opts innate immune cells for tumor promotion (8). Abundant studies have given the importance of immune compositions in TME that can dynamically regulate cancer progression and influence therapeutic outcomes, which has made TME a promising therapeutic target (9–11). Tumor immune microenvironment (TIME) refers to the highly-heterogenous immune context in TME, and great attention has been drawn on understanding its potential role in tumorigenesis. Though ICI therapy has exhibited astonishing efficacy because of the high immunogenicity in SKCM (12), not all patients can be benefited. What’s more, the available tumor staging system is inadequate for a qualified screening of patients who are suitable to accept ICI therapy. Thus, it is necessary to explore novel biomarkers and to understand their roles in the TIME of SKCM, which helps to uncover the potential biology background behind SKCM.

Single-cell RNA-sequencing (scRNA-seq) provides unprecedented opportunities to deconvolve immune system heterogeneity by uncovering novel distinct immune cell subsets, characterizing stochastic heterogeneity within a cell population and building developmental ‘trajectories’ for immune cells (13). This technique can overcome the limitations of traditional RNA-sequencing methods. Another classic population-based RNA-sequencing approach (bulk RNA-seq) is also important in deciphering genome-wide transcriptome variations (14, 15), and it may mask the transcriptional trends of distinct subpopulations with the most abundant cell types or states (16). The organic combination of scRNA-seq and bulk RNA-seq has been applied in studying onco-immunology (17). For example, Joanito I et al. (18) used it to identify two epithelial tumor cell states and refine the consensus molecular classification of colorectal cancer. Kang B et al. (19) revealed key features of the gastric tumor microenvironment through it. Gong L et al. (20) used it to reveal the stromal dynamics and tumor-specific characteristics in the microenvironment of nasopharyngeal carcinoma. Tumor heterogeneity and prognosis-related signatures have been explored with the integrative combination of the two approaches in uveal melanoma (21, 22). However, there is a lack of excavation of the TME in SKCM using scRNA-seq along with bulk RNA-seq. Besides, machine learning is an else indispensable tool, leveraging sophisticated algorithms in processing big, heterogeneous data automatically, professional at prediction problems by revealing useful patterns (23, 24). With the development of bioinformatics, machine learning has become a routine tool for assessing the risk and treatment needs of specific patients. At present, Lasso-Cox is the mainstream algorithm used for generating massive prognosis signatures (25, 26). However, limitations of relying on a single machine learning algorithm may hinder its ability to deliver optimal clinical care to patients, because of the uncertainty to ensure whether the data information of the current algorithm is sufficiently employed, let alone to compare whether the results have reached the population optimal solution. Such inadequate practices can lead to potential overtreatment or undertreatment. Integrated approaches based on various algorithm combinations can fit a consensus model in predicting prognosis, which means the generated model could provide a more personalized evaluation methods to implement clinical decisions.

In the present study, we innovatively integrated afore-mentioned methods. A novel prognostic signature was described and validated to be a potential biomarker. Moreover, as scRNA-seq enables inference of cell-cell communication between tumor cells and their microenvironment (27), we also probed into the profiles of communication networks in SKCM and depicted the specific markers and the indispensable cell–monocyte. The results were confirmed to provide insights in deciphering TME and unveil biological mechanism in SKCM.

## Materials and methods

### Collection of SKCM sample data

The scRNA-seq dataset was obtained with accession number GSE115978 from the Gene Expression Omnibus (GEO) database. Immune cells in it were extracted according to cell markers defined in the original study and 2106 cells were included from 16 pre-immunotherapy patients (28). Raw mapped reads were normalized with counts per million (CPM) and analyzed with quality control using the “Seurat” R package from the normalized. The “FindVariableFeatures” function was performed on the scaled data to screen out the top 2000 genes with higher intercellular coefficient of variation for the downstream analysis (29, 30). The Bulk RNA-seq datasets (TCGA-SKCM, GSE65904 and GSE54467) were acquired from the TCGA and GEO databases respectively. The HTSeq-FPKM gene expression data and related clinical information of SKCM patients were downloaded from the TCGA database as the training group. We enrolled 446 samples whose follow-up time was greater than 0 days with full expression information. For further verification, we adopted the same inclusion criteria. The GSE65904 dataset included 210 SKCM patients, and the GSE54467 dataset included 71 SKCM patients. Via the “Combat” algorithm of the R package “sva”, the possibility of batch effects caused by abiotic bias among the TCGA-SKCM, GSE65904 and GSE54467 datasets was correspondingly reduced (31). It should be noted that both TCGA and GEO databases are open to the public, free of charge. Therefore, this study strictly complies with the data extraction policy of the databases and does not require ethical review and approval from the ethics committee.

### ScRNA sequencing and cell-cell interaction analysis

Principal components analysis (PCA) was conducted on the expression matrix of variable genes. The “FindClusters” function was used with a 0.8 resolution to identify clusters. Also, we performed the “RunTSNE” function to accomplish the dimensionality reduction and visualization processes of t-Distributed Stochastic Neighbor Embedding (t-SNE). The “singleR” package was applied and the cluster-specific markers (log2 |Fold Change| threshold of 1 and FDR threshold of 0.05) were recognized by “FindAllMarkers” function to automatically re-annotate all immune-cell clusters. Immune cells were annotated using the Monaco Immune Database in the Celldex package (1.3.0) (32). The “CellChat” R package (1.1.3) was utilized to infer the immune cell-immune cell communication in the tumor microenvironment (TME) of SKCM on the basis of receptor-ligand interactions (33). The linking numbers were calculated and the communication probability was collected to compute communication networks. The interacting times and total strength of interactions between two arbitrary cell groups were visually displayed. Scatter plots were drawn in order to visualize the dominant sender (signal source) and receiver cells (target) in 2D space, which could help identify the largest contributor signals to the efferent or afferent signals in immune cell sets. We adopted a pattern recognition approach, the global communication model, to discern the way how multiple immune cell types and signaling pathways work in coordination. The “select” function was applied to infer the quantity of patterns, which was comprehensively determined according to two indexes, Cophenetic and Silhouette based on the “non-negative matrix factorization (NMF)” R package (34).

### Signature derived from machine learning-based integrative approaches

Considering the unquestioned importance of monocytes to cell communication, we exploited the monocyte-related signature (MRS). Warranting validations were conducted to make sure MRS has satisfying accuracy, stability and repeatability. We integrated up to 10 machine learning algorithms including random survival forest (RSF), elastic network (Enet), Lasso, Ridge, stepwise Cox, CoxBoost, partial least squares regression for Cox (plsRcox), supervised principal components (SuperPC), generalized boosted regression modeling (GBM), and survival support vector machine (survival-SVM). Upon these methods, a consensus model was produced. Altogether 101 algorithm combinations were performed to match prediction models based on the leave-one-out cross-validation (LOOCV) framework. The TCGA-SKCM dataset was set as a training dataset, the GSE65904 and GSE54467 datasets were set as external validation datasets. Further, the concordance index (C-index) in each pattern in all validation datasets was calculated (35). In total, eighty-seven genes with higher expression level in monocytes than in other immune cells based on scRNA were included in the analysis (log2| Fold Change| threshold of 2 and FDR threshold of 0.05). In line with included gene expression levels from different patterns, we used the linear combination function of each pattern to calculate risk scores. The combined pattern pair from the two external validation datasets with the highest average C-index value was ultimately considered as the most superior one.

### Verifying the accuracy of the signature

After determining the best pattern pairs, we set the median value of the risk scores based on the training dataset as the cutoff to divide patients in both training and validation datasets into either high- or low-risk group. Kaplan-Meier (KM) survival analysis and log-rank test were used on the two groups via the “survival” and “survminer” R packages. Receiver operating characteristic (ROC) curves were depicted to evaluate the accuracy of this signature. What’s more, we performed both the univariate and multivariate cox analysis to prove the independence of the signature. Time-dependent C-index was applied to compare the efficacy of this signature versus traditional clinical variables. In addition, decision curve analysis (DCA) was conducted to judge if the signature can benefit patients in clinical practice. The specific or preponderant cell type that the hub genes from MRS are expressed on were further verified in three independent single-cell datasets (GSE123139, GSE120575, GSE72056) using the “RunUMAP” function.

### Immune microenvironment analysis

Seven different software were utilized to quantify the abundance of immune infiltration of SKCM patients, compare differences of the abundance between the high- and low-risk group, and calculate the Pearson correlations between analysis scores and immune cell contents (36–42). Furthermore, we employed the single sample gene set enrichment analysis (ssGSEA) enrichment scores to assess immune functions. Also, differences of the functions between the high- and low-risk group were then compared using the “wilcox” test (43). Additionally, Thorsson V et al. (44) rendered immunogenomics analyses of more than 10,000 cancers, identifying six immune subtypes that encompass multiple cancer types and are assumed to define immune response patterns influencing prognosis. Four types consist in the TCGA-SKCM, namely wound healing, IFN-gamma dominant, inflammatory and lymphocyte depleted. Subsequently, we focused on the distribution of each subtype in the high- and low-risk group.

### Gene expression, pathway activity and transcriptional heterogeneity analyzes

On account of the training dataset, we compared expression differences of immune checkpoints, costimulatory molecules, chemokines and HLA related genes between the high- and low-risk group. The Molecular Signatures Database (MSigDB) is the most broadly used and comprehensive resource of >10,000 annotated gene sets for use with GSEA software, divided into Human and Mouse collections (45). Nine categories (H, C1-C9) are embodied in Human collections. We chose C2-C8 gene sets to conduct the gene set enrichment analysis (GSEA) for a thorough exposition in differential pathway activities between the high- and low-risk group. To expound on transcriptomics differences a bit further, we analyzed regulon activities of 18 kinds of transcription factors to speculate differential regulation modes in the high- and low-risk group (46).

### Identifying hub genes in the signature

It’s typically believed that if two gene products have similar functions, then terms annotated by them in the Gene Ontology (GO) tree would be close to each other and have high semantic similarity too. The “mgeneSim” function was used to gauge similarity by calculating geometric means of molecular functions and cellular components. Furthermore, we evaluated the importance of each gene by calculating its average similarity to other genes in the signature (47). In addition, we expounded hub gene expression in immune and nonimmune cells in ten SKCM single-cell datasets with the aid of tumor immune single-cell hub (TISCH) database (48, 49). On a final note, the Human Protein Atlas (HPA) database was used to verify whether the expression of hub genes in SKCM was different from that in normal skin at the protein level.

### Statistical analysis

All data processing, statistical analysis, and plotting were performed in R 4.2.0 software. Correlations between two continuous variables were assessed by Pearson’s correlation coefficients. Differential analysis was realized via the wilcox test. P < 0.05 was regarded as statistically significant.

## Results

### T cells and B cells are main cellular components in immune microenvironment of SKCM

**Figure 1:**
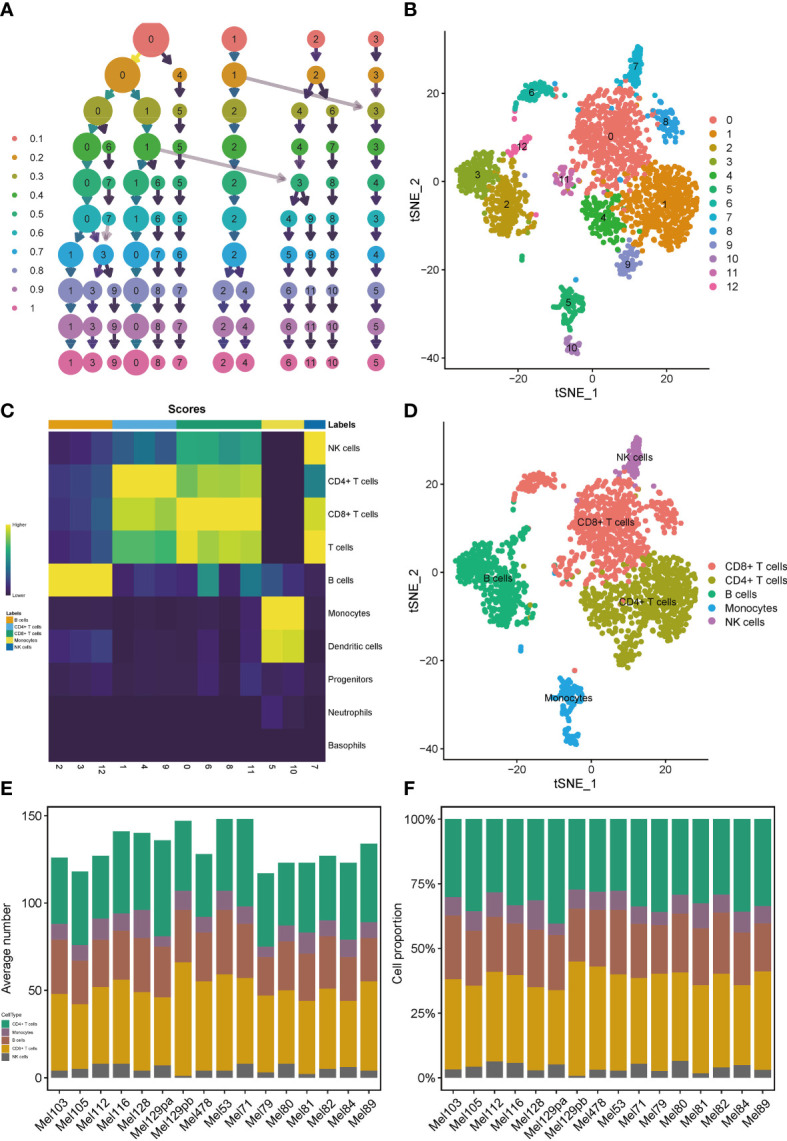
Profiles of immune cells in the TIME of SKCM on the scRNA transcriptome level. Clustering tree for the immune cells clustered using various resolution parameters and the best resolution was 0.8 **(A)**. The t-SNE algorithm helped visualize 13 various cell clusters **(B)**. 7 kinds of immune cells were annotated in the plotScoreHeatmap **(C)**. 5 types of immune cells were annotated as the main labels of the cluster **(D)**. T and B cells were the major compositions of the TIME in SKCM **(E, F)**.

We applied the scRNA-seq dataset (GSE115978) and selected 16 samples from untreated patients for further investigations. Strict quality control measures were taken to obtain 2106 immune cells from predetermined samples, which had 23686 different features. After that, we used the t-SNE algorithm on those 2186 cells to achieve dimension reduction and unsupervised clustering. To decide a desirable resolution parameter for cell clustering, we produced a cluster tree using various resolution values. It was noticed that along with the increase of the resolution, clusters weren’t intertwined much. Therefore, we chose 0.8 as the best resolution because maximum fork clusters were observed (**Figure 1A**). From the t-SNE algorithm, 13 various cell clusters were revealed (**Figure 1B**). Using the “singleR” function, 7 kinds of immune cells were annotated, and the “plotScoreHeatmap” function showed the scores of all cells in all reference labels to check the confidence of the predictive labels throughout the dataset (**Figure 1C**). Among all immune cells, 5 types were annotated as main labels of the cluster. That is, the 0, 6, 8, 11 cell clusters were annotated as CD8+T cells, the 1, 4, 9 cell clusters were annotated as CD4+T cells, the 2, 3 cell clusters were annotated as B cells, the 5, 10 cell clusters were annotated as monocytes and dendritic cells and the 7-cell cluster was annotated as NK cells (**Figure 1D**). It is interesting to note that, the number and proportion of the five main types of immune cells showed a high degree of similarity among 16 samples, suggesting that T and B cells were the major compositions of the SKCM immune microenvironment (**Figures 1E, F**).

### Monocytes are major contributors to both incoming and outgoing signals in immune communication networks

**Figure 2:**
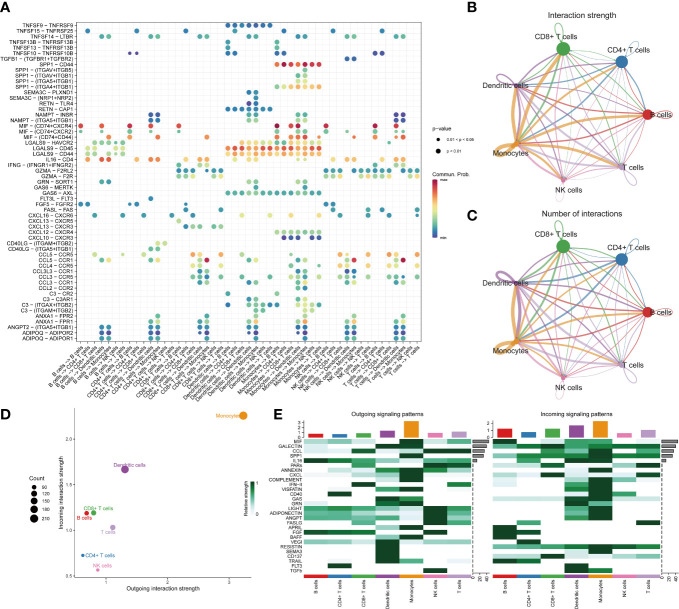
The landscape of immune cell-cell communication. The bubble chart shows overexpressed ligand–receptor interactions. Bubble size represents P value generated by the permutation test, and the color represents the possibility of interactions **(A)**. The circle diagrams show the interaction strength **(B)** and number **(C)** between immune cells. The dot plot shows dominant senders and receivers. The X and Y axes are the total outgoing or incoming communication probabilities associated with each cell group, respectively. The size of the dots is positively related to the number of inferred links (both outgoing and incoming) associated with each cell block. The colors of the dots represent different cell groups **(D)**. The heatmap shows the efferent or afferent contributions of contributions of all signals to different groups of immune cells **(E)**.

**Figure 3:**
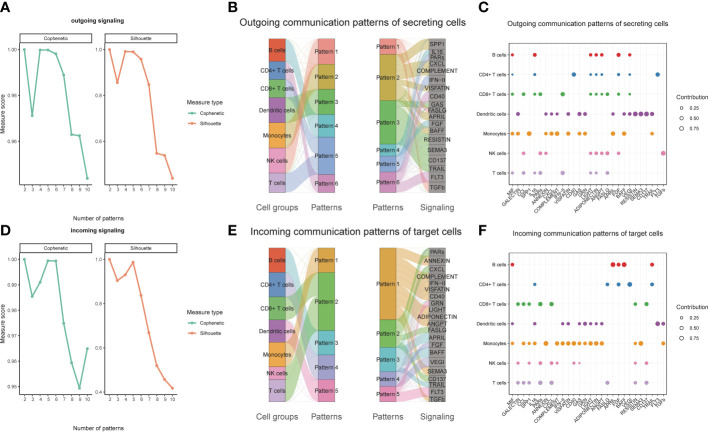
Identifying patterns of outgoing and incoming signals. Numbers of outgoing **(A)** and incoming **(D)** signals based on the Cophenetic and Silhouette indexes. The sankey diagram shows the relations between cell groups and outgoing communication patterns, outgoing communication patterns and signals **(B)**. And relations between cell groups and incoming communication patterns, incoming communication patterns and signals **(E)**. The dot plot shows the contributions of different signals to cell groups in the outgoing communication patterns **(C)**, and incoming communication patterns **(F)**.

We observed over-expressed ligands or receptors and their interactions in seven immune cell groups to identify interactions between immune cells (**Figure 2A**). Circle diagrams showed the interaction times and general strengths of interactions (proportion) between any two cell groups to visualize the integrated cell communication networks. Compared to other immune cells, monocytes were found to contribute the most to both incoming and outgoing signals in immune communication networks (**Figures 2B–D**). Different immune cell groups had obviously different contributive signals on the incoming and outgoing signals (**Figure 2E**). Then the cophenetic and silhouette indexes were combined to recognize 6 efferent and 5 afferent patterns (**Figures 3A, D**). Also, the incoming and outgoing signals were cell-specific. Notably, incoming signals of T cells, CD8+ T cells and NK cells bore similarity (**Figures 3B, E**). At last, **Figures 3C, F** displayed how much diverse signals in efferent and afferent patterns contributed to various cell groups.

### MRS exhibits robust and stable DSS predictive performances

**Figure 4:**
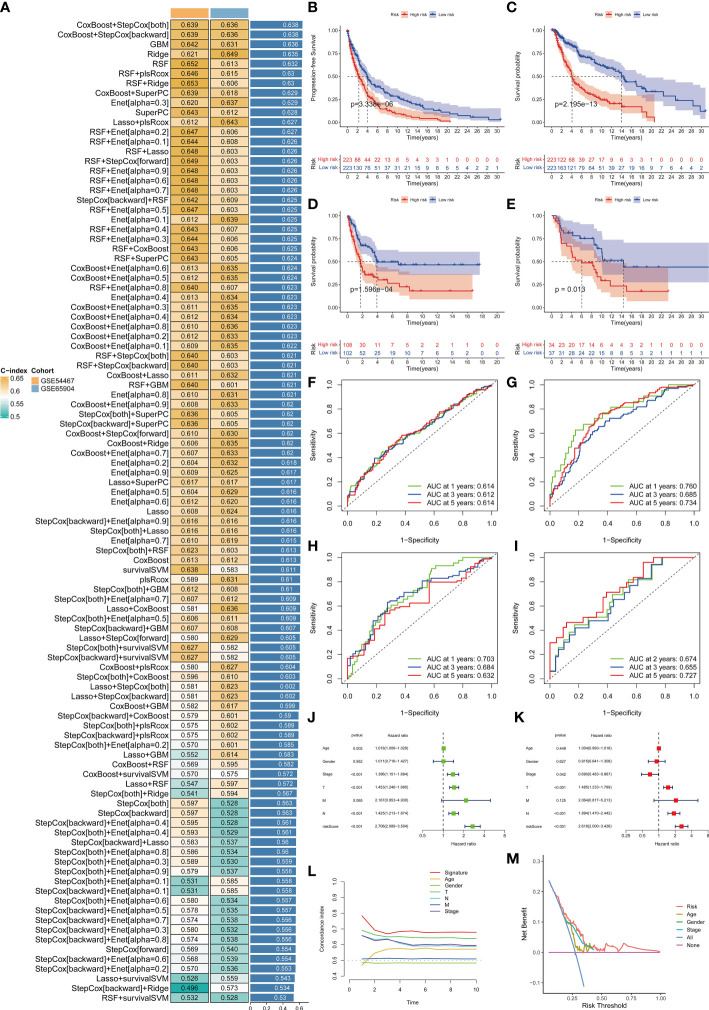
Establishment and validations of a consensus MRS via the machine learning-based integrative procedure. A total of 101 types of prediction models using the LOOCV framework and further calculated the C-index of each model in all validation datasets **(A)**. KM curves of PFS **(B)** and DSS **(C)** between the two risk groups in the training dataset TCGA-SKCM. KM curves of DSS in GSE65904 **(D)** and GSE54467 **(E)** datasets. ROC curves show the 1-, 3- and 5- year PFS in the TCGA-SKCM **(F)** and DSS in the TCGA-SKCM, GSE65904 and GSE54467 datasets **(G–I)**. Univariate **(J)** and multivariate Cox regression analysis **(K)** illustrated that the MRS could be used as an independent prognostic factor for SKCM patients (P< 0.001). Time dependent C-index curves **(L)**, DCA curves **(M)** show the MRS has the good potency in predicting prognosis of SKCM patients.

**Figure 5:**
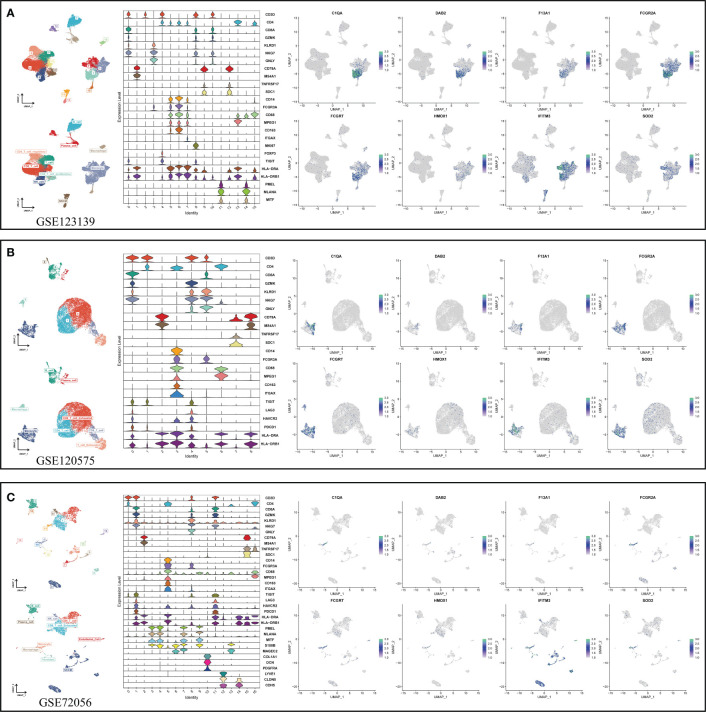
Using three single-cell datasets GSE123139 **(A)**, GSE120575 **(B)**, and GSE72056 **(C)** to verify eight MRS-gene expression location in different cell types. UMAP respectively displays dimension reduction clustering of the dataset. The violin map displays characteristic genes and cell annotation.

In view of the dominant position of monocytes in cell communication, 87 biomarkers in the TCGA-SKCM that are specifically highly expressed in monocytes compared with other immune cells were fitted to 101 prediction models by the LOOCV framework. The C-index of each model was calculated in all validation datasets. An interesting fact was noticed that the best model combination was CoxBoost and stepCox (both) with the highest average C-index (0.638) (**Figure 4A**). Ultimately, an 8-gene monocyte-related signature (MRS) was accordingly established. In the training dataset TCGA-SKCM, we found that the low-risk group owned a relatively longer progression-free survival (PFS) (**Figure 4B**). The high-risk group had a significantly lower disease-specific survival (DSS) in the training dataset (**Figure 4C**), external validation datasets GSE65904 (**Figure 4D**) and GSE54467(**Figure 4E**). Besides, the area under curve (AUC) values of the 1-, 3- and 5- year PFS (**Figure 4F**) and DSS (**Figures 4G–I**) identified by the MRS proved that MRS was a potent prediction tool with stability and strength. MRS had satisfactory specificity and sensitivity. Samples in GSE54467 with DSS within 1 year were too few, hence we chose to evaluate the AUC value of the 2-year DSS. Univariate Cox regression analysis showed that MRS, age, stage, T staging and N staging had close relation to DSS (**Figure 4J**). And the multivariate Cox regression analysis showed that MRS could be treated as an independent prognostic factor for SKCM patients (P< 0.001) (**Figure 4K**). This time-dependent C-index indicated that MRS outperformed conventional clinical variables (**Figure 4L**). Speaking of DCA, it explained that MRS could exactly benefit patients in contrast with conventional clinical variables (**Figure 4M**). All these indicators declared that MRS was stable and robust in the training queue. The classification of different risk groups could be repeated and verified in two independent validation datasets which manifested that MRS was hardly a spurious finding due to technique artifacts, chances or deviations of the sample eligibility criteria in TCGA. Moreover, the cell type that eight MRS-genes were expressed most intensively on was confirmed as monocyte in three single-cell external datasets (GSE123139 (**Figure 5A**), GSE120575 (**Figure 5B**), GSE72056 (**Figure 5C**), which further proved that MRS was stable and repeatable.

### Transcriptome-defined subclasses are biologically distinct and immune infiltration is statistically associated with a more favorable prognosis

**Figure 6:**
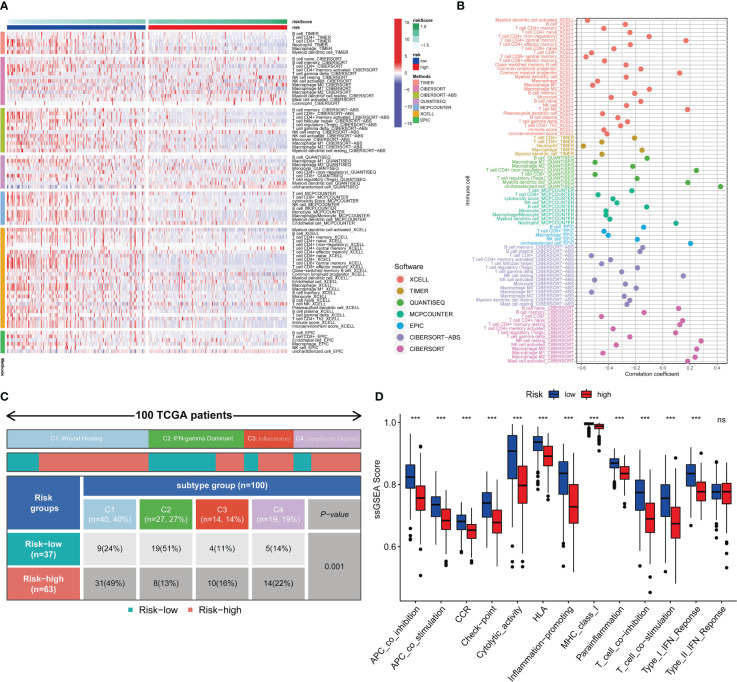
Immune infiltration profiles. Seven immune infiltration software exhibit different numbers of immune cells between the high- and low-risk group **(A)**. The bubble plot shows the correlation between different immune cells and risk scores **(B)**. The block diagram shows the proportion of the high- and low-risk samples in four TCGA-SKCM representative immune subtypes **(C)**. Comparisons of ssGSEA scores between the high- and low-risk group **(D)**. *** means P<0.001.

**Figure 7:**
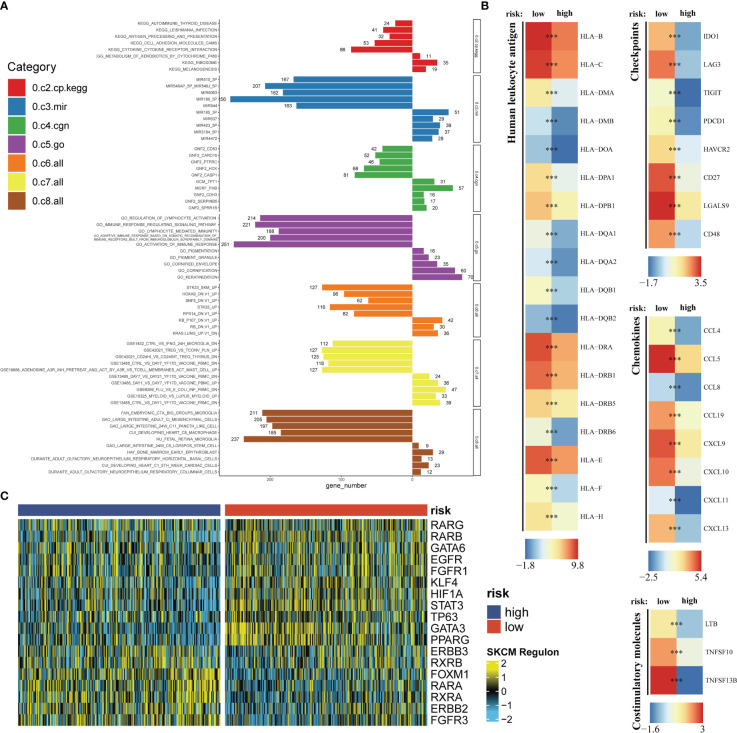
Exploration of the potential risk mechanism. The bar charts show GSEA results in seven gene sets **(A)**. Differential expressions of HLA, immune checkpoints, chemokines and costimulatory molecules between the high- and low-risk group **(B)**. The heatmap shows the cell-regulon activity profiles between the high- and low-risk group **(C)**. *** means P<0.001.

Seven immune infiltration algorithms exerted consistence in which the high-risk group was always infiltrated with less immune cells (**Figure 6A**). Risk scores were significantly positively related to the cell contents of lymphocytes and M1 macrophages (**Figure 6B**). Among immune subtypes of SKCM, we observed that in the low-risk group, there were significantly more patients with IFN-gamma Dominant subtype, but less patients with Lymphocyte Depleted subtype (**Figure 6C**). Besides, ssGSEA results consistently showed that the low-risk group had better immune functions (**Figure 6D**). Immune filtration statistically correlated with better prognosis. The pathway analyzes on the seven datasets vigorously confirmed biological uniqueness of the high- and low-risk group. In the low-risk group, lymphocyte activation, antigen presentation and other related pathways were activated. While in the high-risk group, melanogenesis, keratinization and other related pathways were significantly enriched (**Figure 7A**). HLA, immune checkpoints, chemokines and costimulatory molecules were highly expressed in the low-risk group (**Figure 7B**). Moreover, the cell-regulon activity profiles encompassing 18 types of transcription factors highlighted the possible differential accommodative patterns between the high- and low-risk group (**Figure 7C**).

### IFITM3 has been identified as the core gene in MRS with its high expression in SKCM

**Figure 8:**
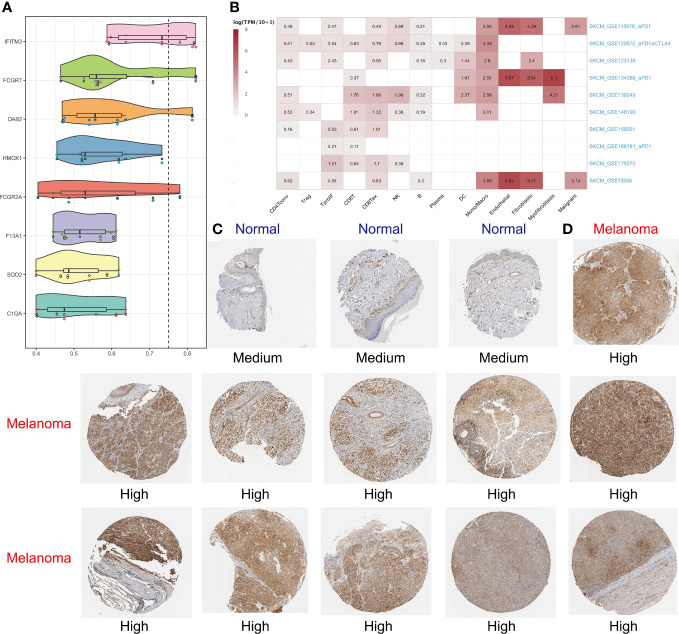
Identifying IFITM3 as the most important gene. The “mgeneSim” function revealing the key gene IFITM3 in the MRS **(A)**. Based on the TISCH database to locate the expression situations of IFITM3 in immune and nonimmune cells in all ten SKCM single-cell datasets **(B)**. The immunohistochemical staining shows IFITM3 expression at protein level in normal and melanoma tissues **(C, D)**.

Using the “mgeneSim” function, we uncovered the key gene IFITM3 in MRS (**Figure 8A**). We employed the TISCH database to locate the expression situations of IFITM3 in immune and nonimmune cells in all ten SKCM single-cell datasets. It was found that IFITM3 not only was highly expressed in mononuclear macrophages, but also in those nonimmune and melanoma cells in the microenvironment (**Figure 8B**). Learning from the immunohistochemical data in the HPA database, we discovered that the expression of IFITM3 in SKCM at protein level was also higher than that in normal skin (**Figures 8C, D**). To sum up, all these results provided corroborative evidence for the exploration value of IFITM3 in the future study of SKCM.

## Discussion

Due to the high rate of metastasis, invasiveness, and annually increasing morbidity, skin cutaneous melanoma (SKCM) is regarded as one of the most lethal skin malignancies globally (50). Conventional histopathology is the mainstay of the diagnosis of SKCM (51), immunohistochemistry and molecular testing have also been introduced to assist with diagnosis (52). Generally speaking, the present staging system for SKCM refers to the 8th edition of TNM classification issued by the American Joint Committee on Cancer (AJCC) in 2017 (53). After the diagnosis confirmed, timely and appropriate staging is of utmost significance, with which an accurate prognostic prediction along with an individualized treatment protocol shall be then provided (54). However, this traditional staging method uses a limited range of parameters equally for all patients, irrespective of clinical context (55) and person-specific heterogeneity. Therefore, sometimes there are limitations on proper treatments and the prognosis may be estimated imprecisely. The emergence of immune checkpoint inhibitors (ICIs) therapy, has made milestone advances in the treatment of SKCM in the last decade, benefiting thousands of SKCM patients and their survival has been prolonged (56, 57). However, only a subset of patients can benefit from ICI therapy. Further still, immune-related toxicities can be a more dangerous dilemma where up to 55% of the patients in the ipilimumab and nivolumab combination trail experienced grade 3 or 4 toxicities (58). As a result, development of a robust biomarker or gene signature to predict response and/or resistance and clinical outcomes is in dire need in SKCM patient management.

In this research, sophisticated, integrated multi-parameter analysis was conducted. We used PCA and t-SNE to cluster massive immune cells, utilizing the “singleR” to annotate them and eventually specific markers of each type of immune cell were identified. Significant specificity existed in the afferent and efferent signals of every immune cell. Monocytes, however, was discovered as a dominant contributor to immune cell-cell communications. Based on the LOOCV framework, we further screened out monocyte-specific markers, on which 101 combination patterns derived from 10 machine learning methods were fitted to ultimately establish a consensus monocyte-related signature (MRS). MRS was subsequently validated in two independent datasets, and the result showed that the best combination pattern consisted of CoxBoost and stepCox (both). MRS was then proved to have negative impact on DSS and PFS, namely the high-risk group identified by the MRS possessed relatively lower DSS and PFS. Our MRS had an excellent and stable performances in clinical use. It was an independent, superior index compared to other traditional clinical variables, and could be served as a good complement to them and their limitations shall be offset in a certain degree. The time-dependent C-index and DCA showed that MRS was a prominent prognosis prediction tool in contrast with those clinical variables, and can benefit patients in an actual way. What’s more, its repeatability was also verified on two external independent queues.

Patients in the high- and low-risk group showed significant biological distinctness too. Hot and cold tumors, are an informal notion to reflect tumor immunogenicity, and the former is highly infiltrated (59). The very reverse, cold tumors is characterized by the lack or low presence of lymphocytes in the TME, resulting in non-responsiveness to ICI therapy (60). Therefore, recent studies have concentrated on the possibility and actuality to turn cold to hot tumors which shows the dynamic changes in the TIME (61), in order to enhance the efficacy of ICI therapy. Notably, the low-risk samples demonstrated phenotypes of hot tumors, infiltrated with abundant lymphocytes and M1 macrophages, significantly enriched with immune-related pathways, and expressed highly of immune-related molecules. This consistently supported that the patients in the low-risk group may be more prone to respond to ICI therapy.

The MRS was composed of 8 genes (C1QA, DAB2, F13A1, FCGR2A, FCGRT, HMOX1, IFITM3, SOD2). Among the 8 hub genes, some have been demonstrated as prognostic biomarkers of cancers already. For example, FCGR2A has been experimentally validated as a positive factor for OS in SKCM (62). Heme Oxygenase 1 (HMOX1) is also regarded as a tumorigenesis-related regulator and is being explored also as a therapeutic target (63). After a comprehensive assessment of the relative importance of each gene, interferon induced transmembrane protein (IFITM3) was recognized as the one with a strong academic exploring value. IFITM3 belongs to the family of interferon induced antiviral proteins. IFITM3 is a well-studied gene in multiple diseases, and has close relation to cancers because it overexpresses in various types. Min J et al. once testified IFITM3’s promotion influences on hepatocellular carcinoma (HCC) invasion and metastasis by regulating MMP9 through p38/MAPK signaling (64). Also in HCC, IFITM3 was demonstrated to facilitate proliferation by upregulating c-myc expression via the ERK1/2 signal pathway (65). Moreover, an investigation led by Chu PY et al. has revealed that the IFITM3 can expand malignant progression, promote cancer stemness and chemoresistance of gastric cancer by targeting MET/AKT/FOXO3/c-MYC axis (66). In addition, high expression of IFITM3 represents adverse prognosis in acute myeloid leukemia (67), and it can cause B-cell malignancies by motivating PI3K pathway (68). Considering that IFITM3 participates in diverse signaling pathways that are likely to cause for oncogenesis and tumor development, it is deemed as a new therapeutic biomarker. Nevertheless, the potential role of IFITM3 in SKCM has not been fully understood yet. In the single-cell research, it is common to respectively study immune cells, nonimmune cells, and tumor cells, to avoid the situation where important information of life beings are mutually obscured by different types of cells. Our research aimed at the immune cell, hence we further evaluated the expression of IFITM3 in nonimmune and tumor cells based on the 10 single-cell datasets in the TISCH database. And the results showed that IFITM3 was highly expressed in the fibroblasts, myofibroblasts, endothelial cells and tumor cells. Via the HPA database, again IFITM3 was proved to express highly in SKCM at the protein level than in normal skins. The synthesis findings helped verify that IFITM3 is of great exploring values and more studies of it as a novel biomarker are needed.

The MRS can be duplicated via some basic PCR-based detection methods, making MRS an attractive tool for clinical transformation and implementation. But it is important to admit that our research has certain limitations as well. To begin with, this study was retrospective. The data and corresponding clinical information were obtained from public accessible TCGA and GEO databases, pretty limited sample size, absence of partial treatment and recurrence records and other artificial errors may potentially distort the findings. Secondly, since the role of IFITM3 in SKCM was not clear, more real-world studies enrolling more tumor specimens and more experiments *in vitro* or *in vivo* should be performed in the future to reveal its actual function. Finally, the current algorithm was based on transcriptome analysis. In the future, the exploration from a global perspective is needed on the law of organism’s life activity. It’s believed that multi-omics integration analysis can promote the deep understanding of life processes and physiological mechanisms, and improve the stability and accuracy of the algorithm to make it gradually perfect, because it provides more features for learning. Additionally, deep learning, as a special kind of machine learning, will automatically find the features that are important for classification, while in machine learning we have to provide these features manually. Therefore, developing new deep learning algorithms and combining multi-omics data can be a powerful and promising tool to help us improve clinical outcomes for individual SKCM patients.

## Conclusions

Our study is the first to establish an 8-gene monocyte-related signature based on abundant machine learning methods. Through adequate validations, the signature has been proved to have stability and strength as a promising predictive biomarker and therapeutic target for SKCM patients. Also, the IFITM3 gene is identified from the signature, and its potential exploring values have been preliminarily confirmed, which may give new inspirations in future clinical use.

## Data availability statement

The original contributions presented in the study are included in the article/**Supplementary Material**. Further inquiries can be directed to the corresponding authors.

## Author contributions

YL and HZ designed the research plan, performed bioinformatics analysis, wrote the manuscript. YM, YS and XW finalized the manuscript. SL, DH and SS supervised the research progress and revised the manuscript. All authors contributed to the article and approved the submitted version.

## Glossary

**Table:**

SKCM	skin cutaneous melanoma
GEO	gene expression omnibus
MRS	monocyte-related signature
DSS	disease specific survival
PFS	progression free survival
HLA	human leukocyte antigen
ICIs	immune checkpoint inhibitors
TME	tumor microenvironment
TIME	tumor immune microenvironment
scRNA-seq	single-cell RNA sequencing
CPM	counts per million
TCGA	the cancer genome atlas
PCA	principal component analysis
t-SNE	t-Distributed Stochastic Neighbor Embedding
NMF	non-negative matrix factorization
RSF	random survival forest
Enet	elastic network
plsRcox	partial least squares regression for Cox
SuperPC	supervised principal components
GBM	generalized boosted regression modeling
survival-SVM	survival support vector machine
LOOCV	leave-one-out cross-validation
ROC	receiver operator characteristic curve
DCA	decision curve analysis
ssGSEA	single sample gene set enrichment analysis
MsigDB	Molecular Signatures Database
GSEA	gene set enrichment analysis
GO	Gene Ontology
TISCH	tumor immune single-cell hub
IFITM3	interferon induced transmembrane protein
AJCC	American Joint Committee on Cancer
HMOX1	Heme Oxygenase 1
HCC	hepatocellular carcinoma

## References

[1] DE Gruijl FR Armstrong BK. Cutaneous melanoma: sheep in wolves clothing? Anticancer Res (2022) 42(10):5021–5. doi: 10.21873/anticanres.16010 36191966

[2] Orzan OA Şandru A Jecan CR. Controversies in the diagnosis and treatment of early cutaneous melanoma. J Med Life (2015) 8(2):132–41. PMC4392104 25866567

[3] Zuluaga-Sepúlveda MA Arellano-Mendoza I Ocampo-Candiani J. Actualización en el tratamiento quirúrgico del melanoma cutáneo primario y metastásico [Update on surgical treatment of primary and metastatic cutaneous melanoma]. Cir Cir (2016) 84(1):77–84. doi: 10.1016/j.circir.2015.06.020 26277601

[4] Garbe C Amaral T Peris K Hauschild A Arenberger P Bastholt L. European Consensus-based interdisciplinary guideline for melanoma. part 2: treatment - update 2019. Eur J Cancer (2020) 126:159–77. doi: 10.1016/j.ejca.2019.11.015 31866016

[5] Leonardi GC Falzone L Salemi R Zanghì A Spandidos DA Mccubrey JA. Cutaneous melanoma: from pathogenesis to therapy (Review). Int J Oncol (2018) 52(4):1071–80. doi: 10.3892/ijo.2018.4287 PMC5843392 29532857

[6] Longvert C Saiag P. Actualités dans le mélanome cutané [Melanoma update]. Rev Med Interne (2019) 40(3):178–83. doi: 10.1016/j.revmed.2018.11.005 30527396

[7] Anderson NM Simon MC. The tumor microenvironment. Curr Biol (2020) 30(16):R921–5. doi: 10.1016/j.cub.2020.06.081 PMC8194051 32810447

[8] Hinshaw DC Shevde LA. The tumor microenvironment innately modulates cancer progression. Cancer Res (2019) 79(18):4557–66. doi: 10.1158/0008-5472.CAN-18-3962 PMC6744958 31350295

[9] Xiao Y Yu D. Tumor microenvironment as a therapeutic target in cancer. Pharmacol Ther (2021) 221:107753. doi: 10.1016/j.pharmthera.2020.107753 33259885 PMC8084948

[10] Bejarano L Jordāo MJC Joyce JA. Therapeutic targeting of the tumor microenvironment. Cancer Discov (2021) 11(4):933–59. doi: 10.1158/2159-8290.CD-20-1808 33811125

[11] Wu T Dai Y. Tumor microenvironment and therapeutic response. Cancer Lett (2017) 387:61–8. doi: 10.1016/j.canlet.2016.01.043 26845449

[12] Ralli M Botticelli A Visconti IC Angeletti D Fiore M Marchetti P. Immunotherapy in the treatment of metastatic melanoma: current knowledge and future directions. J Immunol Res (2020) 2020:9235638. doi: 10.1155/2020/9235638 32671117 PMC7338969

[13] Papalexi E Satija R. Single-cell RNA sequencing to explore immune cell heterogeneity. Nat Rev Immunol (2018) 18(1):35–45. doi: 10.1038/nri.2017.76 28787399

[14] Chen G Ning B Shi T. Single-cell RNA-seq technologies and related computational data analysis. Front Genet (2019) 10:317. doi: 10.3389/fgene.2019.00317 31024627 PMC6460256

[15] Ziegenhain C Vieth B Parekh S Reinius B Guillaumet-Adkins A Smets M. Comparative analysis of single-cell RNA sequencing methods. Mol Cell (2017) 65(4):631–643.e4. doi: 10.1016/j.molcel.2017.01.023 28212749

[16] Slovin S Carissimo A Panariello F Grimaldi A Bouché V Gambardella G. Single-cell RNA sequencing analysis: a step-by-Step overview. Methods Mol Biol (2021) 2284:343–65. doi: 10.1007/978-1-0716-1307-8_19 33835452

[17] Kuksin M Morel D Aglave M Danlos FX Marabelle A Zinovyev A. Applications of single-cell and bulk RNA sequencing in onco-immunology. Eur J Cancer (2021) 149:193–210. doi: 10.1016/j.ejca.2021.03.005 33866228

[18] Joanito I Wirapati P Zhao N Nawaz Z Yeo G Lee F. Single-cell and bulk transcriptome sequencing identifies two epithelial tumor cell states and refines the consensus molecular classification of colorectal cancer. Nat Genet (2022) 54(7):963–75. doi: 10.1038/s41588-022-01100-4 PMC9279158 35773407

[19] Kang B Camps J Fan B Jiang H Ibrahim MM Hu X. Parallel single-cell and bulk transcriptome analyses reveal key features of the gastric tumor microenvironment. Genome Biol (2022) 23(1):265. doi: 10.1186/s13059-022-02828-2 36550535 PMC9773611

[20] Gong L Kwong DL Dai W Wu P Li S Yan Q. Comprehensive single-cell sequencing reveals the stromal dynamics and tumor-specific characteristics in the microenvironment of nasopharyngeal carcinoma. Nat Commun (2021) 12(1):1540. doi: 10.1038/s41467-021-21795-z 33750785 PMC7943808

[21] Gao G Deng A Liang S Liu S Fu X Zhao X. Integration of bulk RNA sequencing and single-cell RNA sequencing to reveal uveal melanoma tumor heterogeneity and cells related to survival. Front Immunol (2022) 13:898925. doi: 10.3389/fimmu.2022.898925 35865532 PMC9294459

[22] Zhang X Qiu J Huang F Han P Shan K Zhang C. Construction and verification of a hypoxia-related nine-gene prognostic model in uveal melanoma based on integrated single-cell and bulk RNA sequencing analyses. Exp Eye Res (2022) 223:109214. doi: 10.1016/j.exer.2022.109214 35981602

[23] Cho YR Kang M. Interpretable machine learning in bioinformatics. Methods (2020) 179:1–2. doi: 10.1016/j.ymeth.2020.05.024 32479800

[24] Goecks J Jalili V Heiser LM Gray JW. How machine learning will transform biomedicine. Cell (2020) 181(1):92–101. doi: 10.1016/j.cell.2020.03.022 32243801 PMC7141410

[25] Zhang G Sun J Zhang X. A novel cuproptosis-related LncRNA signature to predict prognosis in hepatocellular carcinoma. Sci Rep (2022) 12(1):11325. doi: 10.1038/s41598-022-15251-1 35790864 PMC9256635

[26] Feng ZH Liang YP Cen JJ Yao HH Lin HS Li JY. m6A-immune-related lncRNA prognostic signature for predicting immune landscape and prognosis of bladder cancer. J Transl Med (2022) 20(1):492. doi: 10.1186/s12967-022-03711-1 36309694 PMC9617388

[27] Bridges K Miller-Jensen K. Mapping and validation of scRNA-Seq-Derived cell-cell communication networks in the tumor microenvironment. Front Immunol (2022) 13:885267. doi: 10.3389/fimmu.2022.885267 35572582 PMC9096838

[28] Jerby-Arnon L Shah P Cuoco MS Rodman C Su MJ Melms JC. A cancer cell program promotes T cell exclusion and resistance to checkpoint blockade. Cell (2018) 175(4):984–997.e24. doi: 10.1016/j.cell.2018.09.006 30388455 PMC6410377

[29] Satija R Farrell JA Gennert D Schier AF Regev A. Spatial reconstruction of single-cell gene expression data. Nat Biotechnol (2015) 33(5):495–502. doi: 10.1038/nbt.3192 25867923 PMC4430369

[30] Hao Y Hao S Andersen-Nissen E Mauck WM 3rd Zheng S Butler A. Integrated analysis of multimodal single-cell data. Cell (2021) 184(13):3573–3587.e29. doi: 10.1016/j.cell.2021.04.048 34062119 PMC8238499

[31] Johnson WE Li C Rabinovic A. Adjusting batch effects in microarray expression data using empirical bayes methods. Biostatistics (2007) 8(1):118–27. doi: 10.1093/biostatistics/kxj037 16632515

[32] Aran D Looney AP Liu L Wu E Fong V Hsu A. Reference-based analysis of lung single-cell sequencing reveals a transitional profibrotic macrophage. Nat Immunol (2019) 20(2):163–72. doi: 10.1038/s41590-018-0276-y PMC6340744 30643263

[33] Jin S Guerrero-Juarez CF Zhang L Chang I Ramos R Kuan CH. Inference and analysis of cell-cell communication using CellChat. Nat Commun (2021) 12(1):1088. doi: 10.1038/s41467-021-21246-9 33597522 PMC7889871

[34] Gaujoux R Seoighe C. A flexible r package for nonnegative matrix factorization. BMC Bioinf (2010) 11:367. doi: 10.1186/1471-2105-11-367 PMC2912887 20598126

[35] Liu Z Liu L Weng S Guo C Dang Q Xu H. Machine learning-based integration develops an immune-derived lncRNA signature for improving outcomes in colorectal cancer. Nat Commun (2022) 13(1):816. doi: 10.1038/s41467-022-28421-6 35145098 PMC8831564

[36] Becht E Giraldo NA Lacroix L Buttard B Elarouci N Petitprez F. Estimating the population abundance of tissue-infiltrating immune and stromal cell populations using gene expression [published correction appears in genome biol. 2016 Dec 1;17 (1):249]. Genome Biol (2016) 17(1):218. doi: 10.1186/s13059-016-1070-5 27908289 PMC5134277

[37] Newman AM Liu CL Green MR Gentles AJ Feng W Xu Y. Robust enumeration of cell subsets from tissue expression profiles. Nat Methods (2015) 12(5):453–7. doi: 10.1038/nmeth.3337 PMC4739640 25822800

[38] Aran D Hu Z Butte AJ. xCell: digitally portraying the tissue cellular heterogeneity landscape. Genome Biol (2017) 18(1):220. doi: 10.1186/s13059-017-1349-1 29141660 PMC5688663

[39] Li T Fan J Wang B Traugh N Chen Q Liu JS. TIMER: a web server for comprehensive analysis of tumor-infiltrating immune cells. Cancer Res (2017) 77(21):e108–10. doi: 10.1158/0008-5472.CAN-17-0307 PMC6042652 29092952

[40] van Veldhoven CM Khan AE Teucher B Rohrmann S Raaschou-Nielsen O Tjønneland A. Physical activity and lymphoid neoplasms in the European prospective investigation into cancer and nutrition (EPIC). Eur J Cancer (2011) 47(5):748–60. doi: 10.1016/j.ejca.2010.11.010 21159506

[41] Tamminga M Hiltermann TJN Schuuring E Timens W Fehrmann RS Groen HJ. Immune microenvironment composition in non-small cell lung cancer and its association with survival. Clin Transl Immunol (2020) 9(6):e1142. doi: 10.1002/cti2.1142 PMC7291326 32547744

[42] Finotello F Mayer C Plattner C Laschober G Rieder D Hackl H. Molecular and pharmacological modulators of the tumor immune contexture revealed by deconvolution of RNA-seq data [published correction appears in genome med. 2019 jul 29;11(1):50]. Genome Med (2019) 11(1):34. doi: 10.1186/s13073-019-0638-6 31126321 PMC6534875

[43] Barbie DA Tamayo P Boehm JS Kim SY Moody SE Dunn IF. Systematic RNA interference reveals that oncogenic KRAS-driven cancers require TBK1. Nature (2009) 462(7269):108–12. doi: 10.1038/nature08460 PMC2783335 19847166

[44] Thorsson V Gibbs DL Brown SD Wolf D Bortone DS Ou Yang TH. The immune landscape of cancer [published correction appears in immunity. 2019 Aug 20;51(2):411-412]. Immunity (2018) 48(4):812–830.e14. doi: 10.1016/j.immuni.2018.03.023 29628290 PMC5982584

[45] Liberzon A Birger C Thorvaldsdóttir H Ghandi M Mesirov JP Tamayo P. The molecular signatures database (MSigDB) hallmark gene set collection. Cell Syst (2015) 1(6):417–25. doi: 10.1016/j.cels.2015.12.004 PMC4707969 26771021

[46] Lu X Meng J Su L Jiang L Wang H Zhu J. Multi-omics consensus ensemble refines the classification of muscle-invasive bladder cancer with stratified prognosis, tumour microenvironment and distinct sensitivity to frontline therapies. Clin Transl Med (2021) 11(12):e601. doi: 10.1002/ctm2.601 34936229 PMC8693439

[47] Han Y Yu G Sarioglu H Caballero-Martinez A Schlott F Ueffing M. Proteomic investigation of the interactome of FMNL1 in hematopoietic cells unveils a role in calcium-dependent membrane plasticity. J Proteomics (2013) 78:72–82. doi: 10.1016/j.jprot.2012.11.015 23182705

[48] Szklarczyk D Morris JH Cook H Kuhn M Wyder S Simonovic M. The STRING database in 2017: quality-controlled protein-protein association networks, made broadly accessible. Nucleic Acids Res (2017) 45(D1):D362–8. doi: 10.1093/nar/gkw937 PMC5210637 27924014

[49] Sun D Wang J Han Y Dong X Ge J Zheng R. TISCH: a comprehensive web resource enabling interactive single-cell transcriptome visualization of tumor microenvironment. Nucleic Acids Res (2021) 49(D1):D1420–30. doi: 10.1093/nar/gkaa1020 PMC7778907 33179754

[50] Schadendorf D van Akkooi ACJ Berking C Griewank KG Gutzmer R Hauschild A. Melanoma [published correction appears in lancet. 2019 Feb 23;393(10173):746]. Lancet (2018) 392(10151):971–84. doi: 10.1016/S0140-6736(18)31559-9 30238891

[51] Bobos M. Histopathologic classification and prognostic factors of melanoma: a 2021 update. Ital J Dermatol Venerol (2021) 156(3):300–21. doi: 10.23736/S2784-8671.21.06958-3 33982546

[52] Wilson ML. Histopathologic and molecular diagnosis of melanoma. Clin Plast Surg (2021) 48(4):587–98. doi: 10.1016/j.cps.2021.05.003 34503719

[53] Gershenwald JE Scolyer RA Hess KR Sondak VK Long GV Ross MI. Melanoma staging: evidence-based changes in the American joint committee on cancer eighth edition cancer staging manual. CA Cancer J Clin (2017) 67(6):472–92. doi: 10.3322/caac.21409 PMC5978683 29028110

[54] Papageorgiou C Apalla Z Manoli SM Lallas K Vakirlis E Lallas A. Melanoma: staging and follow-up. Dermatol Pract Concept (2021) 11(Suppl 1):e2021162S. doi: 10.5826/dpc.11S1a162S 34447611 PMC8366306

[55] Rashid S Tsao H. Recognition, staging, and management of melanoma. Med Clin North Am (2021) 105(4):643–61. doi: 10.1016/j.mcna.2021.04.005 34059243

[56] Achkar T Tarhini AA. The use of immunotherapy in the treatment of melanoma. J Hematol Oncol (2017) 10(1):88. doi: 10.1186/s13045-017-0458-3 28434398 PMC5402170

[57] Cuevas LM Daud AI. Immunotherapy for melanoma. Semin Cutan Med Surg (2018) 37(2):127–31. doi: 10.12788/j.sder.2018.028 30040090

[58] Buchbinder EI Flaherty KT. Biomarkers in melanoma: lessons from translational medicine. Trends Cancer (2016) 2(6):305–12. doi: 10.1016/j.trecan.2016.05.003 28741528

[59] Galon J Bruni D. Approaches to treat immune hot, altered and cold tumours with combination immunotherapies. Nat Rev Drug Discov (2019) 18(3):197–218. doi: 10.1038/s41573-018-0007-y 30610226

[60] Ochoa de Olza M Navarro Rodrigo B Zimmermann S Coukos G. Turning up the heat on non-immunoreactive tumours: opportunities for clinical development. Lancet Oncol (2020) 21(9):e419–30. doi: 10.1016/S1470-2045(20)30234-5 32888471

[61] Hu R Han Q Zhang J. STAT3: a key signaling molecule for converting cold to hot tumors. Cancer Lett (2020) 489:29–40. doi: 10.1016/j.canlet.2020.05.035 32522692

[62] Zhong F Liu J Gao C Chen T Li B. Downstream regulatory network of MYBL2 mediating its oncogenic role in melanoma. Front Oncol (2022) 12:816070. doi: 10.3389/fonc.2022.816070 35664780 PMC9159763

[63] Chau LY. Heme oxygenase-1: emerging target of cancer therapy. J BioMed Sci (2015) 22(1):22. doi: 10.1186/s12929-015-0128-0 25885228 PMC4380252

[64] Min J Feng Q Liao W Liang Y Gong C Li E. IFITM3 promotes hepatocellular carcinoma invasion and metastasis by regulating MMP9 through p38/MAPK signaling. FEBS Open Bio (2018) 8(8):1299–311. doi: 10.1002/2211-5463.12479 PMC6070650 30087833

[65] Min J Hu J Luo C Zhu J Zhao J Zhu Z. IFITM3 upregulates c-myc expression to promote hepatocellular carcinoma proliferation via the ERK1/2 signalling pathway. Biosci Trends (2020) 13(6):523–9. doi: 10.5582/bst.2019.01289 31852866

[66] Chu PY Huang WC Tung SL Tsai CY Chen CJ Liu YC. IFITM3 promotes malignant progression, cancer stemness and chemoresistance of gastric cancer by targeting MET/AKT/FOXO3/c-MYC axis. Cell Biosci (2022) 12(1):124. doi: 10.1186/s13578-022-00858-8 35941699 PMC9361616

[67] Liu Y Lu R Cui W Pang Y Liu C Cui L. High IFITM3 expression predicts adverse prognosis in acute myeloid leukemia. Cancer Gene Ther (2020) 27(1-2):38–44. doi: 10.1038/s41417-019-0093-y 30923336

[68] IFITM3 enhances PI3K pathway signaling to promote b-cell malignancies. Cancer Discov (2021) 11(1):12. doi: 10.1158/2159-8290.CD-RW2020-168 33218971

