# Supplementary material for: Bulk and single-cell RNA-sequencing analyses along with abundant machine learning methods identify a novel monocyte signature in SKCM
Source: Front Immunol. 2023 May 25;14:1094042. doi: 10.3389/fimmu.2023.1094042 (PMC10248046; doi:10.3389/fimmu.2023.1094042)
Supplement: Supplementary file 1 [file DataSheet_1.pdf]

Signature generated from the combinations of 10 machine learning algorithms

Warranting validations were conducted to make sure MRS has satisfying accuracy, stability and ease of interpretation. The 10 machine learning algorithms refer to random survival forest (RSF), elastic network (Enet), Lasso, Ridge, stepwise Cox, CoxBoost, partial least squares regression for Cox (plsRcox), supervised principal components (SuperPC), generalized boosted regression modeling (GBM), and survival support vector machine (survival-SVM). The MRS generation process was as follows: (i) Based on the leave-one-out cross-validation (LOOCV) framework in the TCGA-SKCM cohort, a combination of 101 algorithms was performed on 87 monocyte characteristics to fit the prediction model. (ii) All models were tested in two validation datasets (GSE65904 and GSE54467). (iii) For each model, the Harrell concordance index (C-index) was calculated for validated datasets, and the model with the highest C-index was considered optimal.

More detailed information is described briefly below:

Lasso, stepwise Cox, CoxBoost, and RSF can be used to accomplish the feature selection step. Therefore, these algorithms were applied comprehensively in order to establish a consensus model. As a result, there were up to 101 algorithm combinations conducted to fit the prediction models based on the leave-one-out cross-validation (LOOCV) framework. We performed the first attempt of the signature in the TCGA-SKCM dataset. The conducted the randomForestSRC package to generate the RSF model. RSF had two parameters ntree and mtry, and the former represented the quantity of trees in the forest and the latter meant the quantity of randomly selected variables for splitting at each node. A grid-search was implemented on the two parameters via the LOOCV framework. Forming all pairs of (ntree, mtry), the pair with the best C-index value then would be identified as the optimal parameter pair. The Lasso, Enet, and Ridge were conducted using the glmnet package. The regularization parameter, λ, was determined by LOOCV, and the L1-L2 trade-off parameter, α, was set to 0-1 (interval =0.1). The stepwise Cox model was conducted using the survival package. A stepwise algorithm based on the Akaike information criterion (AIC) was adopted, and the direction mode of stepwise search was set as "both", "backward", and "forward", respectively. The CoxBoost model was conducted using the CoxBoost package, that is utilized to fit a Cox proportional hazards model by componentwise likelihood-based boosting. For the CoxBoost model, the LOOCV routine optimCoxBoostPenalty function was used to first determine the optimal penalty (amount of shrinkage). Once this parameter was determined, the other tuning parameter of the algorithm, namely, the number of boosting steps to perform, was selected using the function cv.CoxBoost. Eventually, the principal routine CoxBoost was used to set dimension of the selected multivariate Cox model. The plsRcox model was implemented through the plsRcox package. The cv.plsRcox function was used to determine the number of required components, and the plsRcox function was applied to fit a partial least squares regression generalized linear model. The SuperPC model, implemented via the superpc package, is a generalization of principal component analysis (PCA) that generates a linear combination of the features or variables of interest that capture the directions of largest variation in a dataset. The superpc.cv function used a form of LOOCV to estimate the optimal feature threshold in supervised principal components. The pre-validation method was used to avoid problems with fitting Cox models to small validation datasets. The GBM model was implemented using the superpc package. With the LOOCV framework, the cv.gbm function selected index for number trees with minimum cross-validation error. And the gbm function was applied to fit the generalized boosted regression model. The survival-SVM model was carried out via the survivalsvm package. The regression approach considers auditing when formulating the inequality constraints of the support vector problem.
